# Optimization of a Novel Method Based on Ultrasound-Assisted Extraction for the Quantification of Anthocyanins and Total Phenolic Compounds in Blueberry Samples (*Vaccinium corymbosum* L.)

**DOI:** 10.3390/foods9121763

**Published:** 2020-11-28

**Authors:** María José Aliaño-González, José Antonio Jarillo, Ceferino Carrera, Marta Ferreiro-González, José Ángel Álvarez, Miguel Palma, Jesús Ayuso, Gerardo F. Barbero, Estrella Espada-Bellido

**Affiliations:** 1 Department of Analytical Chemistry, Faculty of Sciences, Agrifood Campus of International Excellence (ceiA3), IVAGRO, University of Cadiz, 11510 Puerto Real, Cadiz, Spain; mariajose.alianogonzalez@alum.uca.es (M.J.A.-G.); joseantoniojarillo@gmail.com (J.A.J.); ceferino.carrera@uca.es (C.C.); marta.ferreiro@uca.es (M.F.-G.); miguel.palma@uca.es (M.P.); estrella.espada@uca.es (E.E.-B.); 2 Department of Physical Chemistry, Faculty of Sciences, Institute of Biomolecules (INBIO), University of Cadiz, 11510 Puerto Real, Cadiz, Spain; joseangel.alvarez@uca.es (J.Á.Á.); jesus.ayuso@uca.es (J.A.)

**Keywords:** anthocyanins, antioxidants, blueberry, Box–Behnken design, phenolic compounds, response surface methodology, ultra-high-performance liquid chromatography, UHPLC, UV–Vis, *Vaccinium corymbosum* L.

## Abstract

In recent years, consumers’ preference for fruits such as blueberry has increased noticeably. This fact is probably related to their bioactive components such as anthocyanins, phenolic compounds, vitamins, minerals, and tannins that have been found in blueberries by the latest research studies. Both total anthocyanins (TA) and total phenolic compounds (TPC) are known for their multiple beneficial effects on our health, due to their anti-inflammatory, anti-oxidant, and anti-cancer properties. This is the reason why the development of new methodologies for the quality control analysis of raw materials or derived products from blueberry has a great relevance. Two ultrasound-assisted extraction methods (UAE) have been optimized for the quantification of TA and TPC in blueberry samples. The six variables to be optimized were: solvent composition, temperature, amplitude, cycle, extraction solvent pH, and sample/solvent ratio using response surface methodology. The optimized methods have proven to be suitable for the extraction of the TPC and TA with good precision (repeatability and intermediate precision) (coefficient of variation (CV) < 5%) and potentially for application in commercial samples. This fact, together with the multiple advantages of UAE, makes these methods a good alternative to be used in quality control analysis by both industries and laboratories.

## 1. Introduction

Blueberry is a small reddish or bluish-black fruit with a size of around 1.5 cm in diameter that grows in deciduous shrubs from the Ericaceae family [1,2]. Blueberry has been extensively cultivated worldwide with an estimated production of over 600,000 tons per year, being Canada, Chile, China, Spain, USA, and Morocco, among other 30 countries, the major blueberry distributors [3].

Although blueberry is not so often consumed as fresh fruit, it is increasingly common to find it as the main ingredient in jams and pastry preparations or as a secondary ingredient in different candies or desserts (yogurts, cookies, etc.) [4,5,6]. Moreover, recent investigations have proven its high content in anthocyanins, phenolic compounds, vitamins, and minerals. A fact that has caused blueberry to be included in different extracts and numerous food supplements [7].

The Ericaceae family includes nearly 4000 different plant species, where the *Vaccinium* genus is one of the best known and more frequently consumed [8]. The *Vaccinium* genus can be categorized into four main varieties: bilberry (*V. myrtillus*), blueberry (*V. angustifolium, V. ashei,* and *V. corymbosum*), cranberry (*V. macrocarpon* and *V. oxycoccos*), and lingonberry (*V. Vitis-idaea*), with *V. corymbosum* being one of the most cultivated varieties. Its fruit, with a bittersweet and rather pleasant flavor, exhibits very interesting nutritional properties because of its high content in compounds of biological interest such as phenolic compounds, like catechol, coumaric acid, chlorogenic acid, ellagic acid, epicathechin, gallic acid, anthocyanins (cyanidin 3-*O*-galactoside, delphinidin 3-*O*-arabinoside, petunidin 3-*O*-galactoside, malvidin 3-*O*-galactoside, etc.) and flavonols (mainly quercetin derivatives) [9,10,11,12,13]. Culture conditions of *V. corymbosum* have shown a clear influence on its composition, including phenolic compounds. In relation to temperature, different authors have shown that temperatures between 20 and 30 °C ensure the highest phenolic concentration in blueberries, while higher temperatures have adverse impacts on their growth and composition [14,15,16]. In addition, the use of ammonium compounds (RNH_4_^+^) improved the plant performance and the phenolic compounds composition. Similar effects were detected when phytochemicals with acid pH were applied, indicating that acidification is an important mechanism in the composition of blueberry [17,18].

Different research works have proven that blueberry, similarly to other dark-colored berries, contains a high polyphenols concentration, such as total anthocyanins (TA) and phenolic compounds (TPC) [19]. Consequently, blueberry extracts have exhibited beneficial properties for human health that have been associated with the presence of these compounds. They have been confirmed as a considerable advancement for the treatment of cardiovascular diseases [20,21], diabetes [22] or even for the prevention of atherosclerosis [23,24]. In addition, decisive anti-inflammatory activity has been appreciated [25], since they reduce cell death and diminish the morphological criteria that are associated with inflammation in microglia cell cultures. This discovery represents a novel way for the treatment of some neurological diseases [26]. They have also shown a significant activity to decrease oxidative stress (anti-oxidant) with a significant effect against pulmonary arterial hypertension [27], skin damage by UV radiation [28,29] and in the microbial diversity with health benefits [30,31]. Finally, blueberry extracts have exhibited a meaningful activity that favors the treatment of different types of cancer including leukemia [32,33,34].

Therefore, it has been proven that extracts rich in TA and TPC obtained from blueberry are suitable for a considerable number of applications in the nutrition, cosmetics or medicine industries. For this reason, the development of new methodologies that allow the extraction of these compounds from the blueberries in a fast, easy to use and reliable way is of great importance. Several analytical extraction techniques such as maceration [35,36,37], Soxhlet [38,39], pressurized liquid extraction [40,41], microwave-assisted extraction [42,43,44], have been employed to obtain rich anthocyanins and phenolic compound extracts from berries. Among them, ultrasound-assisted extraction (UAE) [45] and pressurized liquid extraction (PLE) [46,47] are the most commonly used for the extraction of both bioactive compounds in blueberries.

UAE has been successfully used for the recovery of TA and TPC. This technique produces the extraction of the organic compounds that can be found in different matrices making use of the energy derived from ultrasounds. Its efficiency is attributed to acoustic cavitation, a phenomenon which consists on the formation, development and collapse of microbubbles on the surface of the solid, which allows the penetration of the solvent into the solid and favors mass transfer processes [48]. In addition, ultrasounds exert a mechanical effect that can contribute to both the release of intracellular material and the desorption of compounds from the solid surface, resulting in higher extraction rates. Finally, the use of a wider temperature range results in an increased production of cavitation bubbles and consequently improved extraction efficiency [49].

In addition, UAE presents some critical advantages, such as its simplicity, low acquisition cost, no specific maintenance requirements and availability in most laboratories. For all these reasons, this technique has been largely applied to the extraction of bioactive compounds from similar matrices such as myrtle [50], açai [51], black chokeberry [52], maqui [53], sesame [54,55,56]. In fact, different researchers has previously employed UAE to study the blueberry composition [57,58,59,60,61,62]. However, as far as the authors are concerned, an exhaustive study (evaluating seven extraction variables) using UAE for the extraction of these bioactive compounds from blueberries has not yet been performed. Different solvents have been applied on UAE of several fruits, with methanol being one of the most employed due to its high effectivity in TA and TPC extraction. It is true that methanol has been classified as class 2 according to FDA (Food and Drug Administration) [63] due to the toxicity and the health consequences when it is consumed. Nevertheless, it is extensively recommended for analytical purposes due to its polarity, viscosity and small size, which makes it very easy to penetrate into cell membranes.

This research is focused on the optimization of two methods based on UAE. Both methods have been combined with spectrometric techniques to confirm that the developed optimized methods produce a successful recovery of TA and TPC from blueberry and, therefore, could be used by industries and laboratories. Following such purposes, repeatability and intermediate precision analyses have been performed and the methods that have been developed have been tested on real samples to demonstrate their applicability.

## 2. Materials and Methods

### 2.1. Blueberry Samples

To optimize the extraction methods, 5 kg of blueberries at their optimal state of maturity (*Vaccinium corymbosum* L. var. Legacy) was collected from a 1.5 ha plantation at the village of Ballota (Asturias-Spain; 43.552571, −6.329105) during the month of August. The sampling was carried out randomly throughout the entire crop plot, picking blueberries from all parts of the plant (upper, middle and lower zone). Afterward, the fresh berries were freeze-dried and crushed by means of a conventional electric grinder and the final matrix was homogenized and kept at −20 °C in a freezer until use.

Once the two methods were optimized, they were tested on commercial samples to ensure their actual suitability for industrial and research analysis. In order to guarantee a diversity of samples, five blueberry (*Vaccinium corymbosum* L.) jams were acquired from three different sources and with different content percentages of blueberry according to the respective product’s label. Their descriptions can be seen in Table 1. The commercial samples were also kept at −20 °C until analysis.

### 2.2. Solvents and Chemical Agents

Methanol (Fisher Scientific, Loughborough, UK), and formic acid (Panreac, Barcelona, Spain), both high-performance liquid chromatography (HPLC) grade, were used for extraction and chromatographic analysis. Ultra-pure water was obtained from a Milli-Q water purifier system from Millipore (Bedford, MA, USA) and hydrochloric acid (Panreac, Barcelona, Spain; “for analysis” grade) was employed to adjust the pH of the solvents for the extraction.

For the anthocyanin quantification, the standard cyanidin chloride was acquired with a purity higher than 95% from Sigma–Aldrich Chemical Co. (St. Louis, MO, USA) whereas for total phenolic compounds analysis, Folin–Ciocalteu reactive (EMD Millipore, Darmstadt, Germany), sodium carbonate anhydrous (Panreac, Barcelona, Spain), and gallic acid standard ≥ 99% (Sigma–Aldrich Chemical Co., St. Louis, MO, USA) were used.

### 2.3. Ultrasound-Assisted Extraction

A UP200S sonifier (200 W, 24 kHz) (Dr. Hielscher. GmbH, Teltow, Germany) equipment was selected to carry out this study. The sonifier included a water bath coupled to a temperature controller (FRIGITERM-10, J.P. Selecta, S.A., Barcelona, Spain) to allow the optimization of this variable during the development of the method.

Six variables were selected for the optimization and three different levels were established for the range of study (low, medium and high): extraction solvent (25–50–75% MeOH in water), temperature of extraction (10–40–70 °C), amplitude (30–50–70% of the maximum amplitude—200 W), cycle (0.2–0.45–0.7 s), pH of the extraction solvent (2–4.5–7) and samples’ mass (g):solvent volume (mL) ratio (0.5:10–0.5:15–0.5:20). Both the selected variables to be optimized and their range of study were chosen according to the existing bibliography and the previous experience of the research group with similar matrices and compounds [51,53,64]. The amount of sample and the time of the experiment were set at 0.5 g and 10 min, respectively, based on similar studies previously completed by our research group [43,65].

In this way, 0.5 g of the sample was weighted and the specific amount of solvents to reach the ratio corresponding to each experiment was added. These solvents had been previously prepared to ensure the right percentage of methanol in water corresponding to each experiment.

The required volume of solution was added to the amount of sample, the extraction probe was put in the mixture and the extraction was carried out under the specific conditions of each experiment. After 10 min, the centrifugation process was realized twice for 5 min at 7500 rpm (9.5 cm orbital radius). The supernatant was moved to a 25 mL volumetric flask and a 0.20 μm nylon syringe filter (Membrane Solutions, Dallas, TX, USA) was employed before the analysis.

### 2.4. Box–Behnken Design

This research was aimed at developing two extraction methods that would achieve the maximum possible recoveries of TA and TPC from freeze-dried blueberry samples. For this purpose, a Box–Behnken design with response surface methodology (BBD-RSM) was used to optimize the six extraction variables. The amount of TA (mg of anthocyanins per g of sample) determined by ultra-high-performance liquid chromatography (UHPLC) was used as the response variable and the TPC (mg of gallic acid equivalents (GAE) per g of sample) determined by the method of Folin–Ciocalteu was considered as the other response variable. The resulting design included a total of 54 extractions including 6 at the central point (Table S1).

The second-order polynomial equation below, which includes all the six variables, was applied to the responses obtained from all the extractions.

Y = *β_0_* + *β*_1 × 1_ + *β*_2_X_2_ + *β*_3_X_3_ + *β*_4_X_4_ + *β*_5_X_5_ + *β*_6_X_6_ + *β*_12_X_1_X_2_ + *β*_13_X_1_X_3_ + *β*_14_X_1_X_4_ + *β*_15_X_1_X_5_ + *β*_16_X_1_X_6_ + *β*_23_X_2_X_3_ + *β*_24_X_2_X_4_ + *β*_25_X_2_X_5_ + *β*_26_X_2_X_6_ + *β*_34_X_3_X_4_ + *β*_35_X_3_X_5_ + *β*_36_X_3_X_6_ + *β*_45_X_4_X_5_ + *β*_46_X_4_X_6_ + *β*_56_X_5_X_6_ + *β*_11_X_1_^2^ + *β*_22_X_2_^2^ + *β*_33_X_3_^2^ + *β*_44_X_4_^2^ + *β*_55_X_5_^2^ + *β*_66_X_6_^2^ (1)

In our equation, Y is the corresponding response, *β*_0_ is the ordinate at the origin; X_1_ (percentage of MeOH in the extraction solvent), X_2_ (the extraction temperature), X_3_ (the ultrasound amplitude), X_4_ (the cycle), X_5_ (the solvent pH), and X_6_ (the ratio of solid sample (g): extraction volume (mL)) are the independent variables; *β*_i_ represents the linear coefficients; *β*_ij_ depicts the cross product coefficients and *βii* shows the quadratic coefficients.

Statgraphic Centurion (version XVII) (Statgraphics Technologies, Inc., The Plains, VA, USA) was the software used to calculate the effects of the extraction variables on the final response, the second-order mathematical model, the surface plots, the optimal levels of the significant variables and the variance of the analysis.

### 2.5. Anthocyanins Identification by UHPLC-Q-ToF-MS

The TA present in the blueberry samples were identified by an ultra-high-performance liquid chromatography (UHPLC) system coupled to quadrupole time-of-flight (Q-ToF-MS) mass spectrometry (XEVO G2, Waters Corp., Milford, MA, USA). The chromatography column employed was a C18 with dimensions of 2.1 mm × 100 mm and a particle size of 1.7 µm (Acquity UPLC BEH C18, Waters). The injection volume was set at 3 μL. The chromatographic conditions used as well as the analysis conditions in the mass spectrometer (Q-ToF-MS) are those described by Aliaño et al. [42].

The identification of the TA present in blueberries was determined by the exact *m*/*z* mass/charge ratio obtained for each of the chromatographic peaks corresponding to anthocyanins. The ratio was compared with other works found in the literature that describe the anthocyanins present in blueberries [66], and by the order of elution of the anthocyanins. A total of 14 anthocyanins were identified in the blueberry extracts; the data regarding each compound and the theoretical and measured mass/charge ratios can be found in Table 2.

### 2.6. Separation and Quantification of Anthocyanins

Once the TA were identified by UHPLC-Q-ToF-MS, its separation and quantification was the next step to be completed. A LaChrom Ultra Elite UHPLC system (VWR Hitachi, Tokyo, Japan) was used for this purpose. This system included an L-2200U autosampler, an L-2160U pump and an L2300 column oven which was set at 50 °C for the analysis. In addition, the UHPLC was equipped with an L-2420U UV–vis Detector that was set at 520 nm for anthocyanin quantification. The anthocyanins were analyzed on a Hitachi LaChrom HaloTM C18 column (100 × 3 mm inside diameter, particle size 2.7 µm).

Acidified water (5% formic acid) was selected as solvent A, while pure methanol was chosen as solvent B, working at a flow rate of 1.0 mL min^−1^. The chromatographic separation was performed by the following gradient method: 0 min, 15% B; 1.50 min, 20% B; 3.30 min, 30% B; 4.80 min, 40% B; 5.40 min, 55% B; 5.90 min, 60% B; 6.60 min, 95% B; 9.30 min, 95% B; 10 min, 15% B.

Cyanidin chloride was used as anthocyanin standard, obtaining the following calibration curve: y = 300,568.88x − 28,462.43. The regression equation and the determination coefficient (R^2^ = 0.9999) were determined by means of Microsoft Office Excel 2010.

The Shapiro–Wilk test and the *t*-test were used to evaluate the normal distribution of the residues, obtaining a W value of 0.8514 (very close to 1) and a *p* value of 0.803 (higher than 0.05), respectively, which demonstrated the normal distribution of the residues.

**Figure 1:**
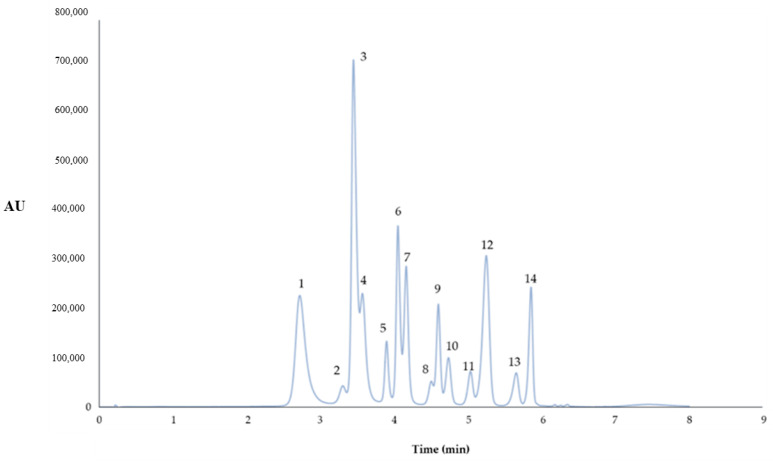
Ultra-high-performance liquid chromatography (UHPLC) chromatogram of the fourteen anthocyanins identified in the blueberry extract (λ = 520 nm). Peak assignment: (1) delphinidin 3-*O*-galactoside; (2) delphinidin 3-*O*-glucoside; (3) cyanidin 3-*O*-galactoside; (4) delphinidin 3-*O*-arabinoside; (5) cyanidin 3-*O*-glucoside; (6) petunidin 3-*O*-galactoside; (7) cyanidin 3-*O*-arabinoside; (8) petunidin 3-*O*-glucoside; (9) peonidin 3-*O*-galactoside; (10) petunidin 3-*O*-arabinoside; (11) peonidin 3-*O*-glucoside; (12) malvidin 3-*O*-galactoside; (13) malvidin 3-*O*-glucoside; (14) malvidin 3-*O*-arabinoside; AU—Absorbance units.

Finally, the detection limit (0.198 mg L^−1^) and the quantification limit (0.662 mg L^−1^) were calculated as was described by Aliaño et al. [42]. The UHPLC chromatogram representing the 14 anthocyanins is shown in Figure 1.

The cyanidin chloride calibration curve was used to quantify the fourteen anthocyanins present in the blueberry extracts, assuming that the different anthocyanins have similar molar attenuation coefficient (ε) and taking into account the molecular weight of each anthocyanin. All the analyses were performed in triplicate and the results were expressed as mg of anthocyanins per g of blueberry. Regression equations of the 14 anthocyanins identified in blueberry extracts are presented in Table 2.

### 2.7. Total Phenolic Content (TPC)

For the analysis of the TPC of the blueberry extracts, the Folin–Ciocalteu methodology was used, as described by Singleton and Rossi in 1965 [66] but with certain modifications suggested by Singleton et al. in 1999 [67] for extracts of vegetables. A solution of gallic acid in methanol at 1000 mg L^−1^ was used as the standard, and methanol dilutions in the range of 0.1–500 mg L^−1^ were used to draw up the standard curve. This procedure has been described in our previous work [42].

The calibration curve obtained for the gallic acid standard was y = 0.0024x − 0.0031. The determination coefficient was R^2^ = 0.9999 while the limit of detection and the limit of quantification were 1.649 mg L^−1^ and 5.498 mg L^−1^, respectively; the limits were calculated as explained in Section 2.6 above. The normal distribution of the residues for the gallic acid standard was also studied; the Shapiro–Wilk test showed a W value of 0.9201 and the *p*-value was 0.762, which indicated the normal distribution of the residues.

### 2.8. Statistical Analysis

As it was previously mentioned, a Box–Behnken design was selected to evaluate the influence of different variables on TA and TPC extraction. For that, the effect of the extraction variables on the final response was studied. The surface plots were evaluated to see how these variables affect the TA and TPC extraction. Once the optimal levels of the significant variables were obtained, a repeatability and intermediate precision study was carried out. Standard deviation and coefficient of variation were the statistical parameters selected to evaluate the precision. Statgraphic Centurion (version XVII) (Statgraphics Technologies, Inc., The Plains, VA, USA) was the software used for the statistical analysis.

## 3. Results

The aim of this research was the optimization of two different methods both based on UAE to find the maximum recovery of bioactive compounds (TA and TPC) from blueberry samples. Thus, they could be used by industries and laboratories for quality control analysis and to ensure the maximum possible compound concentration in both the raw material and their commercially derived products. For this purpose, a BBD-RSM was selected and a total of 54 experiments with specific conditions for each of them were obtained (Table S1). The extractions were performed in duplicate according to these parameters and after that the TA and TPC were measured. The TA and TPC contents were used to determine the optimal conditions to achieve the maximum yields of these compounds. In all the cases, the analytical parameters of repeatability and intermediate precision have been evaluated to guarantee the suitability of the developed methods. Finally, commercial samples were analyzed under the optimum conditions to prove the suitability of the developed methods.

### 3.1. Anthocyanins Optimization

#### 3.1.1. Optimization of the Extraction Method

The extracts obtained from the 54 experiments were analyzed by UHPLC–UV–vis to quantify the 14 anthocyanins previously identified. The individual anthocyanin content was aggregated to determine the total anthocyanin content and the average of the two replicates of the same experiment was used as the response variable.

BBD-RSM was applied to determine the influence from each one of the six variables and their possible interaction on the response variable. On the other hand, the correlation between the real values of the TA and the values predicted from Equation (1) was evaluated, and the differences between the actual and the predicted values were computed as relative prediction error (Table S1). The mean prediction error was 4.79%, with values ranging from 0.02% to 15.84%, which implies a clear influence of the extraction variables on the anthocyanin recoveries and, consequently, the possibility of adjusting them to obtain the maximum anthocyanin recovery from the blueberry samples.

The *t*-test was employed to evaluate the influence of the optimized variables, considering a 95% confidence level, which means that the variables with *p*-values lower than 0.05 were considered influential. Table 3 shows the calculated *p*-values. As it can be seen, the most influential variables were: percentage of methanol in the solvent (*p*-value: 0.0005), the quadratic interaction of the percentage of methanol in the water used as extraction solvent (*p*-value: 0.0268), and the interaction percentage of methanol: amplitude (*p*-value: 0.0295). In linear terms, the percentage of methanol in the extraction solvent had a positive influence, which means that the anthocyanin extractions were more favorable when the percentage of methanol was higher.

**Figure 2:**
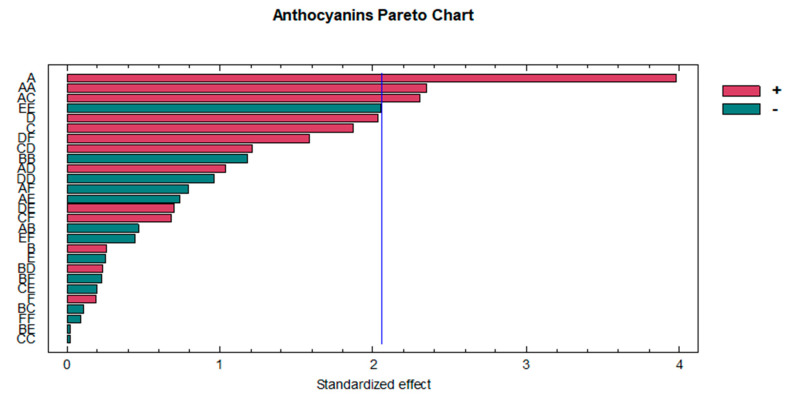
Standardized Pareto chart representing the extraction optimization of total anthocyanins. A: solvent (% MeOH); B: temperature (°C); C: amplitude (%); D: cycle (s); E: pH; F: ratio (mL).

A standardized Pareto chart (Figure 2) was used to graphically represent and examine the influence of the variables and their order of importance. As previously mentioned, the content of methanol in water was the most influential variable for anthocyanin extraction. The solvent composition was expected to be one of the most influential variables according to the previous results by this research team, and based on these, a polarity of the solvent similar to that of anthocyanins would be required to ensure its extraction. The composition of the solvent and the quadratic interaction of the extraction solvent have been previously detected as influential variables also for the extraction of anthocyanins from other similar matrices such as sloe [64], black chokeberry [52] or açai [51]. Furthermore, the relationship between the extraction solvent and the amplitude also turned out to be an influential variable; all of them had a positive coefficient but with a significantly lower influence than that exerted by the extraction solvent.

#### 3.1.2. Optimal Conditions

**Figure 3:**
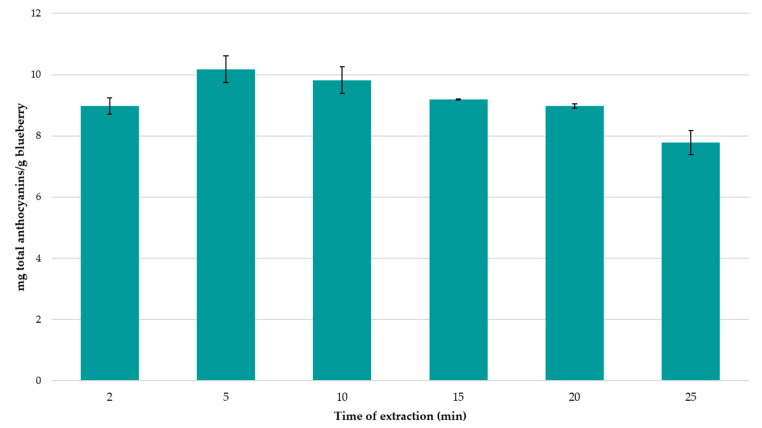
Study of the extraction time to optimize the recovery of anthocyanins from blueberry extracts (*n* = 3). The error bars represent the uncertainty of the measurement in the three replicates.

The use of BBD-RSM allows us to determine the optimal extraction variables to ensure the maximum recovery of TA from the blueberry samples. The optimal conditions were determined at 0.5 g of sample extracted at 34 °C with 20 mL of a solvent containing 74.6% MeOH in water at pH 4. It was detected that the optimal amplitude (70%) and cycle (0.7) values corresponded to the maximum values within the studied range. However, the cycle was not detected as an influential variable and the higher amplitude values caused splashes that resulted in some extract losses and, therefore, an optional increment of both ranges of variables was discarded. Finally, it was expected that relatively low temperature values within the studied range would yield the optimal extraction values, since anthocyanins have shown to be easily degraded under extreme thermal conditions [68]. Other authors have found for bilberry that the extracts after 60 min applying ultrasound had a lower concentration of anthocyanins than the extracts obtained only by conventional maceration [69]. This may be because the sonication process can lead to the formation of free radicals that can enhance polymerization/depolymerization reactions. To see how ultrasound influences our particular extraction conditions, a comparison was made between the total concentration of anthocyanins obtained through the optimal extraction conditions obtained for ultrasound (5 min), with the same optimal extraction conditions, without the application of ultrasound, applying only maceration with magnetic stirring (5 min and 60 min; 300 rpm). The extractions have been carried out in triplicate. Under our extraction conditions, a greater amount of total anthocyanins has been obtained by applying ultrasound, compared to using maceration with magnetic stirring (32.16% for 5 min and 9.42% for 60 min). Although it is true that ultrasound can degrade anthocyanins, by applying ultrasound for a very short period of time, and through cycles, this degradation can be minimized and the positive effects of ultrasound on the extraction of compounds can be optimized. On the other hand, longer ultrasound times present a considerable reduction in the total amount of anthocyanins extracted (Figure 3), so it is recommended to use ultrasound for short periods of time and under optimal extraction conditions.

The optimal conditions were compared with recent research studies on the extraction of anthocyanins from berry matrices using UAE. Bonat Celli et al. [68] investigated the extraction of anthocyanin from haskap berries (*Lonicera caerulea* L.) using UAE. The optimal conditions established were similar to those found for this research, such as the ratio (25:1 (mL g^−1^)), temperature (35 °C), or percentage of methanol (80%). However, the extraction time required was 20 min, which is twice the length of the period measured in this research.

On the other hand, Quiang Cheng et al. [70] and R. Albuquerque et al. [71] studied the extraction of anthocyanins from the fruits of *Rubia sylvatica* Nakai and from Jabuticaba’s epicarp, respectively. Both authors included pH as an influential variable and demonstrated that acid-level pH (3–4) facilitated the extraction of anthocyanins similarly as it was observed in the present research. Finally, Fibigr et al. [72] investigated the optimal amount of açai berry samples for maximum anthocyanin extraction using UAE. Thus, the experiments performed with 0.4–0.7 g samples allowed the maximum recoveries. As it could be observed, the optimal conditions determined for the extraction of anthocyanins from blueberries were in agreement with the results found in the literature.

#### 3.1.3. Extraction Time

Once the optimal values of the six selected extraction variables had been determined, the next step consisted of studying the extraction time and its influence on the TA extraction. For this purpose, the extractions were carried out under optimal conditions but with six different extraction times (2, 5, 10, 15, 20 and 25 min) each one of them performed in triplicate. Thus, eighteen extracts were obtained which were analyzed by UHPLC–UV–vis and the fourteen anthocyanins were quantified. Total anthocyanins were calculated as the sum of the individual anthocyanins. The average concentration of TA for the different experimental times has been represented in Figure 3. As it can be observed, all the experiments achieved a content of anthocyanins above 7 mg per gram of blueberry sample. However, the maximum concentration of anthocyanins (10.18 mg/g) was reached when 5 min of extraction were used, although no significant differences were noticed when compared to the 10 min-extraction experiments. Therefore, for its lesser energy consumption, 5 min was selected as the optimal extraction time. In fact, a decreasing concentration was detected when 15 min-extraction time was applied and this trend continued up to 25 min-extraction time. This fall in anthocyanin concentrations could be closely related to the high susceptibility of anthocyanins to be degraded that had been observed in similar research studies [73] and for this reason the authors do not recommend any extraction processes that require 10 min or a longer time.

#### 3.1.4. Repeatability and Intermediate Precision

Once optimized, to ensure the suitability of the developed method to be used by different industries and/or laboratories, the evaluation of repeatability and intermediate precision was required.

For this purpose, 10 extractions were carried out under optimal conditions on three different consecutive days and a total of 30 extractions were obtained under the optimal conditions that had been established. These extracts were analyzed by UHPLC–UV-vis and the recovery of TA was calculated as explained before. The coefficient of variation (CV) was the analytical factor selected for this evaluation. The CV of the samples taken on the same day were computed for repeatability while, for intermediate precision, this factor was calculated based on the samples taken on the three consecutive days. The values obtained are shown in Table 4. The coefficient of repeatability was 4.17%, while the coefficient of intermediate precision was 4.32%, both of them below 5%.

#### 3.1.5. Re-Extraction Study for Anthocyanins

To check the effectiveness of the extraction method, a re-extraction study of the residues obtained after applying the optimized method was carried out. The residue obtained was subjected to the optimal extraction conditions of the method. This study was carried out in triplicate, obtaining a total amount of anthocyanins less than 5%, so it can be considered a quantitative method.

### 3.2. Phenolic Compounds Optimization

#### 3.2.1. Optimization of the Extraction Method

The extracts obtained from the 54 extractions were also analyzed by Folin–Ciocalteu methodology and the TPC was determined. The response variable used was the average of the replicates of the same experiment.

The values obtained from the analysis (real values) were correlated with the predicted values from Equation (1), and the differences were considered as relative prediction error (Table S1). As can be seen, the average prediction error was 3.84% and ranged from 0.36% up to 13.08%. These results suggest an influence of the variables that had been considered for the study on the TPC extracted from the blueberry samples. The BBD-RSM method was, therefore, applied in order to determine the influence of those variables as well as their possible interaction on the TPC content. This should allow us to determine the optimal conditions to achieve the maximum recovery of TPC.

The *p*-values were calculated according to the *t*-test considering a 95% confidence level, which means that the variables with *p*-values lower than 0.05 were considered influential. In linear terms, the solvent ratio was an influential variable (*p*-value: 0.0013) with positive influence, which means that a higher amount of solvent would result in a greater extraction of TPC. Furthermore, the quadratic interaction of the percentage of methanol in the extraction solvent and the interaction of the percentage of methanol and the cycle revealed *p*-values below 0.05 (*p*-value: 0.0002 and *p*-value: 0.0301) which implies that they were influential variables even though they were not so by themselves. On the other hand, the quadratic interaction of the extraction temperature (*p*-value: 0.0113), the quadratic interaction of the ratio (*p*-value: 0.003) and the interaction between the extraction temperature and the ratio (*p*-value: 0.0419) were also influential variables, although the individual variables were not found to be influential by themselves. The calculated *p*-values can be seen in Table 5.

**Figure 4:**
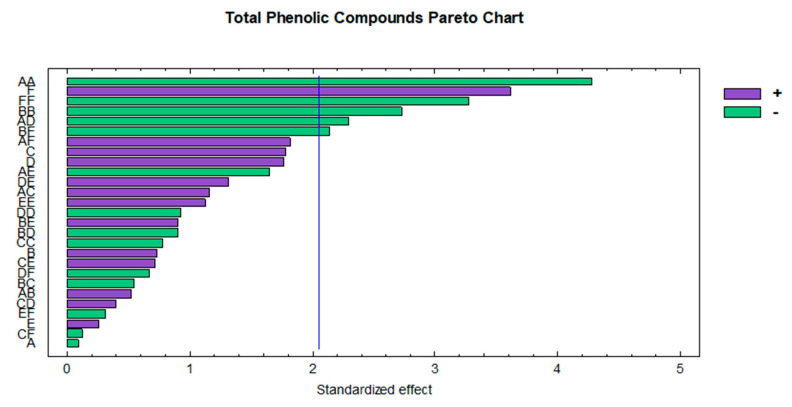
Standardized Pareto chart representing the optimization of total phenolic compounds extraction. A: solvent (% MeOH); B: temperature (°C); C: amplitude (%); D: cycle (s); E: pH; F: ratio (mL).

The standardized Pareto chart (Figure 4) was plotted in order to graphically observe the influence of the variables. It was noted that the quadratic interaction of the methanol percentage was the most influential variable with a negative effect on the extraction of TPC. Recent investigations conducted by our research group on the extraction of TPC from similar matrices such as black chokeberry [52] or myrtle [50] confirmed the influence of methanol percentage on the extraction of these compounds. The solvent ratio was the second most influential variable with a positive effect, as previously mentioned, while the rest of the influential variables (quadratic interaction of the extraction temperature, quadratic interaction of the ratio and the interaction between the extraction temperature and the ratio) showed negative coefficients. Likewise, these quadratic interactions have been observed as influential variables in numerous studies that focused on the extraction of TPC from fruit matrices such as mulberry [65] or açai [40].

#### 3.2.2. Optimal Conditions

Once the influential variables for the extraction of total phenolic compounds, had been identified, BBD-RSM was applied to determine the optimal extraction variables that would ensure the maximum recovery of TPC. The optimal conditions were established at 0.5 g of sample extracted at 33.3 °C with 16 mL of solvent containing 44% MeOH in water at pH 7. Finally, 0.7 cycles at 70% amplitude were selected for the ultrasound system to ensure the maximum extraction of TPC as commented for anthocyanins. Although the optimum pH was set at the maximum value within the range, no higher points were tested since, according to the literature, basic pH could cause the degradation of the phenolic compounds [74,75]. Some similarities were observed with respect to the optimal conditions for the extraction of TA. However, the extraction of TPC was improved when using solvents with a lower percentage of methanol and with a more basic pH than that used for the extraction of TA.

Espada-Bellido et al. [65] carried out an optimization research on the extraction of phenolic compounds from mulberry (*Morus nigra*) pulp using UAE. The optimal conditions were similar to those already determined in this research, namely the solvent pH (7), the ultrasound amplitude (70%), the cycle (0.7) or the ratio (11:1.5 mL g^−1^). However, the percentage of methanol (61%) and the temperature (64 °C) required were higher than those observed in this study; both factors could be closely interrelated to the nature of the sample.

#### 3.2.3. Extraction Time

**Figure 5:**
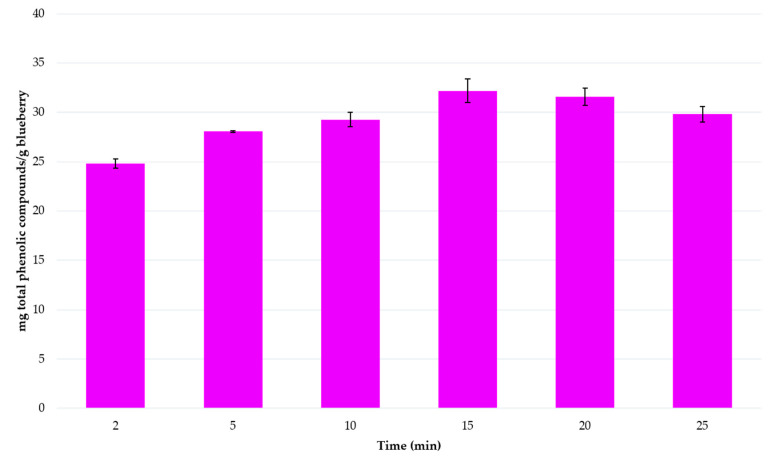
Study of the optimal extraction time study for the recovery of total phenolic compounds from blueberry extracts (*n* = 3). The error bars represent the uncertainty of the measurement in the three replicates.

Similarly, to the research on TA extraction, a study to determine the optimal extraction time for the recovery of the TPC was required. For this purpose, different extractions were performed under the optimal conditions. Six different extraction times were employed (2, 5, 10, 15, 20 and 25 min) in triplicate. The extracts were analyzed by Folin–Ciocalteu methodology and the total content in phenolic compounds was determined. The average of the three replicates under the same conditions was calculated and represented for each extraction time in Figure 5. The maximum total phenolic content (32.18 mg g^−1^) was reached when the extraction time was 15 min, without any significant difference when compared to the extraction from the 20 min experiments. For this reason, the optimal extraction time was established at 15 min. Furthermore, it was observed that after that time the TPC began to decrease. This fact could be closely related to the degradation of the compounds, as it has been observed in similar matrices such as açai [51], sloe [64] or blackberries [43].

#### 3.2.4. Repeatability and Intermediate Precision

The optimized method for the extraction of total phenolic compounds was also evaluated for reliability. For this purpose, in the same way as for the anthocyanins, 10 extractions were carried out on the same day and 10 more extractions on 2 consecutive days. The extracts were analyzed by Folin–Ciocalteu methodology and the total phenolic compounds content was determined. The CV was calculated for the samples analyzed on the same day and on different days. The results can be seen in Table 4.

The method’s repeatability exhibited a CV of 3.97% while for its intermediate accuracy the CV obtained was 3.85%; both of them are below 5%, which confirms that the optimized methods are reliable and they could be applied in different industries/laboratories with no relevant differences to be expected between their results.

#### 3.2.5. Re-Extraction Study for Total Phenolic Compounds

As previously carried out for TA, to check the effectiveness of the extraction method, a re-extraction study of the residues obtained after applying the optimized method was carried out. The residue obtained was subjected to the optimal extraction conditions of the method. This study was carried out in triplicate, obtaining a total amount of phenolic compounds less than 5%, so it can be considered a quantitative method for these compounds.

### 3.3. Application to Real Samples

In the course of this research, two methods based on UAE have been developed. These methods have focused on maximizing the extraction of TA and TPC from blueberry samples. Furthermore, both methods have displayed good repeatability and intermediate accuracy, which means that they could be used both in industrial and research laboratories. Finally, the methods developed were applied to five commercial samples such as jams with blueberry contents (Table 1).

**Figure 6:**
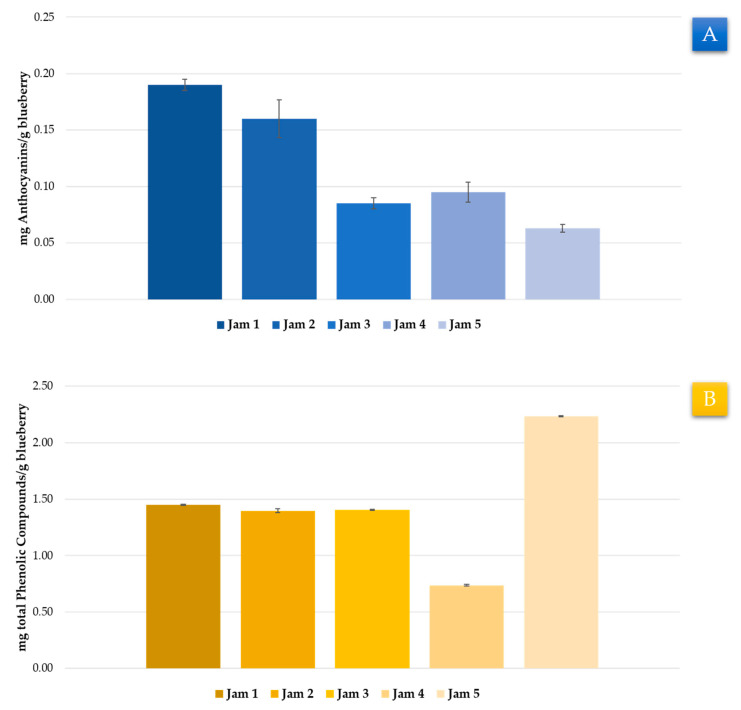
Application of the optimized method to real samples. (**A**) Anthocyanin content in real samples according to the developed method (*n* = 3). (**B**) Total phenolic compounds in real samples according to the developed method (*n* = 3). The error bars represent the uncertainty of the measurement in the three replicates.

With respect to TA, the samples did not undergo any pre-treatment; 0.5 g of the sample was weighed and extracted under the corresponding optimal conditions for TA before being analyzed by UHPLC–UV–vis. All the samples were extracted in triplicate. The TA were calculated as the sum of 14 individual anthocyanins. The average amount from the replicates of the same sample was calculated and can be seen in Figure 6A. The TA content extracted from the real samples was significantly lower than that obtained from the freeze-dried samples, with the sample from Jam 1 being the one with the largest content. Such lower concentration of anthocyanins in the real samples was expected, since the samples in our experiments were plain fresh berries while the jams contained over 50% sugar, which represents a significant dilution of the blueberry content. In addition, the authors suggest a possible degradation of the anthocyanin because of the temperature reached to obtain the jam matrix, which would greatly depend on the elaboration process applied to each sample. In addition, different research works have shown a considerable reduction in anthocyanins in blueberry jams after several months of storage due to the generation of anthocyanin-procynidin polymers [76], which could be another cause of the decrease in TA observed in jam samples.

Regarding the total phenolic compounds, the jam samples (0.5 g) were extracted under optimal conditions for the extraction of total phenolic compounds. The extracts were filtered through a 0.20 μm nylon syringe filter and Folin–Ciocalteu methodology was applied. All the samples were extracted in triplicate. The average concentrations of the replicates were calculated and the results are shown in Figure 6B. It was detected that, similarly to the anthocyanins, the phenolic content was lower in the real samples than the one obtained from the freeze-dried samples. This could be due to the plain fresh berries used in our experiments, while the blueberries in the jam had been mixed with other elements. It could be observed that Jam 5 had a considerably high content of phenolic compounds, which could be closely related to a lower elaboration temperature and, consequently, to a lesser degradation of the compounds of interest.

## 4. Conclusions

In the present research, two methodologies have been developed for the extraction of TA and TPC from blueberries. These methodologies have been based on UAE because of the many advantages associated with this system, such as ease of use, low power consumption, low cost and the fact that it does not require any maintenance while it is available in most laboratories.

A Box–Behnken design has been carried out to obtain the optimal conditions for the TA and TPC extraction from freeze-dried blueberry samples. In addition, an extraction time study was taken into account and it was observed that only 5 and 15 min were required for the TA and TPC extraction, respectively. In addition, both methods have demonstrated good repeatability and intermediate accuracy with a CV below 5%, which confirms their suitability to be applied by different laboratories and industries. Finally, both optimized methods were tested on real jam samples with a varying blueberry content and proved to be perfectly suitable for the intended purposes.

The results obtained and the important advantages associated with ultrasound-assisted extraction, including among others the fact of being a rapid, repeatable, inexpensive and easy-to-use technique, suggest that both the methodologies developed could be easily used by laboratories or industries for quality control analysis to assure the content of anthocyanins and total phenolic compounds either in their raw material or in their intermediate or finished blueberry products.

## Figures and Tables

**Table 1:** Commercial samples acquired to corroborate the validity of the optimized extraction methods.

Commercial Mark	Origin of Production	% Blueberry
Jam 1	Spain	45
Jam 2	Spain	50
Jam 3	Spain	60
Jam 4	Brazil	50
Jam 5	France	65

**Table 2:** Mass information and regression equation data of the 14 anthocyanins identified in blueberry extracts.

Anthocyanins	Molecular Formula	*m*/*z*	Regression Equation	R^2^ *	LOD ** (ppm)	LOQ *** (ppm)
Malvidin 3-*O*-galactoside	C_23_H_25_O_12_	493.1346	y = 170,426.24x − 4292.66	0.9999	0.303	1.012
Malvidin 3-*O*-glucoside	C_23_H_25_O_12_	493.1346	y = 170,426.24x − 4292.66	0.9999	0.303	1.012
Petunidin 3-*O*-galactoside	C_22_H_23_O_12_	479.1190	y = 175,412.35x − 4292.66	0.9999	0.294	0.983
Petunidin 3-*O*-glucoside	C_22_H_23_O_12_	479.1190	y = 175,412.35x − 4292.66	0.9999	0.294	0.983
Delphinidin 3-*O*-galactoside	C_21_H_21_O_12_	465.1033	y = 180,699.41x − 4292.66	0.9999	0.286	0.955
Delphinidin 3-*O*-glucoside	C_21_H_21_O_12_	465.1033	y = 180,699.41x − 4292.66	0.9999	0.286	0.955
Peonidin 3-*O*-galactoside	C_22_H_23_O_11_	463.1240	y = 181,468.74x − 4292.66	0.9999	0.284	0.951
Peonidin 3-*O*-glucoside	C_22_H_23_O_11_	463.1240	y = 181,468.74x − 4292.66	0.9999	0.284	0.951
Malvidin 3-*O*-arabinoside	C_23_H_23_O_11_	463.1240	y = 181,468.74x − 4292.66	0.9999	0.284	0.951
Cyanidin 3-*O*-galactoside	C_21_H_21_O_11_	449.1084	y = 187,132.66x − 4292.66	0.9999	0.276	0.922
Cyanidin 3-*O*-glucoside	C_21_H_21_O_11_	449.1084	y = 187,132.66x − 4292.66	0.9999	0.276	0.922
Petunidin 3-*O*-arabinoside	C_21_H_21_O_11_	449.1084	y = 187,132.66x − 4292.66	0.9999	0.276	0.922
Delphinidin 3-*O*-arabinoside	C_20_H_19_O_11_	435.0927	y = 193,161.98x − 4292.66	0.9999	0.267	0.893
Cyanidin 3-*O*-arabinoside	C_20_H_19_O_10_	419.0978	y = 200,531.32x − 4292.66	0.9999	0.257	0.860

* Coefficient of determination; ** Limit of detection; *** Limit of quantification.

**Table 3:** Analysis of variance of the quadratic model adjusted to the extraction of anthocyanins. A: %MeOH; B: temperature; C: amplitude; D: cycle; E: pH; F: ratio.

Variable	Source	Coefficient	Sum of Squares	Degrees of Freedom	Mean Square	*F*-Value	*p*-Value
	*β*_0_	24.0196	325.9582	27			
A	X_1_	−0.3187	105.9240	1	105.9240	15.8500	0.0005
B	X_2_	0.1353	0.4483	1	0.4483	0.0700	0.7977
C	X_3_	−0.2988	23.3445	1	23.3445	3.4900	0.0729
D	X_4_	−24.0790	27.6705	1	27.6705	4.1400	0.0522
E	X_5_	3.0896	0.4401	1	0.4401	0.0700	0.7995
F	X_6_	−0.1373	0.2400	1	0.2400	0.0400	0.8512
A:A	X_1_^2^	0.0030	36.8281	1	36.8281	5.5100	0.0268
A:B	X_12_	−0.0006	1.4878	1	1.4878	0.2200	0.6410
A:C	X_13_	0.0042	35.4482	1	35.4482	5.3000	0.0295
A:D	X_14_	0.1073	7.1958	1	7.1958	1.0800	0.3090
A:E	X_15_	−0.0108	3.6585	1	3.6585	0.5500	0.4660
A:F	X_16_	−0.0058	4.2195	1	4.2195	0.6300	0.4340
B:B	X_2_^2^	−0.0011	9.2232	1	9.2232	1.3800	0.2507
B:C	X_23_	−0.0002	0.0861	1	0.0861	0.0100	0.9105
B:D	X_24_	0.0290	0.3785	1	0.3785	0.0600	0.8138
B:E	X_25_	−0.0002	0.0053	1	0.0053	0.0000	0.9778
B:F	X_26_	−0.0014	0.3570	1	0.3570	0.0500	0.8190
C:C	X_3_^2^	0.0000	0.0041	1	0.0041	0.0000	0.9805
C:D	X_34_	0.2210	9.7682	1	9.7682	1.4600	0.2375
C:E	X_35_	−0.0037	0.2665	1	0.2665	0.0400	0.8433
C:F	X_36_	0.0044	3.1418	1	3.1418	0.4700	0.4990
D:D	X_4_^2^	−12.4244	6.2022	1	6.2022	0.9300	0.3442
D:E	X_45_	1.0280	3.3025	1	3.3025	0.4900	0.4883
D:F	X_46_	1.1570	16.7331	1	16.7331	2.5000	0.1256
E:E	X_5_^2^	−0.2649	28.1965	1	28.1965	4.2200	0.0501
E:F	X_56_	−0.0326	1.3285	1	1.3285	0.2000	0.6594
F:F	X_6_^2^	−0.0030	0.0596	1	0.0596	0.0100	0.9255
Pure Error			173.7380	26	6.6822		
Total			539.3450	53			

**Table 4:** Repeatability and intermediate precision study for anthocyanins and phenolic compounds recovery from blueberry extracts.

	Repeatability (*n* = 10)	Intermediate Precision (*n* = 30)
Average mg anthocyanins/g blueberry	10.07	10.13
Standard deviation	0.42	0.44
Coefficient of variation (CV) (%)	4.17	4.32
Average mg total phenolic compounds/g blueberry	31.28	30.94
Standard deviation	1.24	1.19
Coefficient of variation (CV) (%)	3.97	3.85

**Table 5:** Analysis of variance of the quadratic model adjusted to the extraction of total phenolic compounds. A: %MeOH; B: temperature; C: amplitude; D: cycle; E: pH; F: ratio.

Variable	Source	Coefficient	Sum of Squares	Degrees of Freedom	Mean Square	*F*-Value	*p*-Value
	*β*_0_	−9.1847		27			
A	X_1_	0.2657	0.0241	1	0.0241	0.0100	0.9227
B	X_2_	0.2620	1.3348	1	1.3348	0.5300	0.4723
C	X_3_	0.0308	7.9465	1	7.9465	3.1700	0.0868
D	X_4_	15.8107	7.8318	1	7.8318	3.1200	0.0890
E	X_5_	−0.9338	0.1785	1	0.1785	0.0700	0.7918
F	X_6_	2.3123	32.7367	1	32.7367	13.0500	0.0013
A:A	X_1_^2^	−0.0034	45.9076	1	45.9076	18.3000	0.0002
A:B	X_12_	0.0004	0.6962	1	0.6962	0.2800	0.6028
A:C	X_13_	0.0013	3.4061	1	3.4061	1.3600	0.2545
A:D	X_14_	−0.1454	13.2132	1	13.2132	5.2700	0.0301
A:E	X_15_	−0.0148	6.8450	1	6.8450	2.7300	0.1106
A:F	X_16_	0.0081	8.2825	1	8.2825	3.3000	0.0808
B:B	X_2_^2^	−0.0015	18.6571	1	18.6571	7.4400	0.0113
B:C	X_23_	−0.0005	0.7503	1	0.7503	0.3000	0.5891
B:D	X_24_	−0.0672	2.0301	1	2.0301	0.8100	0.3766
B:E	X_25_	0.0048	2.0521	1	2.0521	0.8200	0.3741
B:F	X_26_	−0.0080	11.4960	1	11.4960	4.5800	0.0419
C:C	X_3_^2^	−0.0010	1.5125	1	1.5125	0.6000	0.4445
C:D	X_34_	0.0450	0.4050	1	0.4050	0.1600	0.6911
C:E	X_35_	0.0080	1.2800	1	1.2800	0.5100	0.4814
C:F	X_36_	−0.0005	0.0371	1	0.0371	0.0100	0.9042
D:D	X_4_^2^	−7.3422	2.1660	1	2.1660	0.8600	0.3614
D:E	X_45_	1.1820	4.3660	1	4.3660	1.7400	0.1986
D:F	X_46_	−0.3020	1.1401	1	1.1401	0.4500	0.5062
E:E	X_5_^2^	0.0887	3.1619	1	3.1619	1.2600	0.2719
E:F	X_56_	−0.0142	0.2521	1	0.2521	0.1000	0.7538
F:F	X_6_^2^	−0.0647	26.9429	1	26.9429	10.7400	0.0030
Pure Error			65.2306	26	2.5089		
Total			261.2350	53			

## Supplementary Materials

The following are available online at https://www.mdpi.com/2304-8158/9/12/1763/s1, Table S1. Box–Behnken design matrix with the values of the six variables for each experiment and measured and predicted responses (*n* = 2).
