# Supplementary material for: Optimization of a Novel Method Based on Ultrasound-Assisted Extraction for the Quantification of Anthocyanins and Total Phenolic Compounds in Blueberry Samples (Vaccinium corymbosum L.)
Source: Foods. 2020 Nov 28;9(12):1763. doi: 10.3390/foods9121763 (PMC7759891; doi:10.3390/foods9121763)
Supplement: Supplementary file 1 [file foods-09-01763-s001.pdf]

*Article*

# Optimization of a Novel Method Based on Ultrasound-Assisted Extraction for the Quantification of Anthocyanins and Total Phenolic Compounds in Blueberry samples (*Vaccinium corymbosum* L.)

**SUPPLEMENTARY MATERIAL**

**Table S1.** Box-Behnken design matrix with the values of the six variables for each experiment and measured and predicted responses ($n$ = 2).

| | % MeOH | Temperature (°C) | Amplitude | Cycle | pH | Ratio | mg anthocyanins measured /g Blueberry | mg anthocyanins predicted /g Blueberry | Relative error of prediction (%) | mg total Phenolic Compounds measured /g Blueberry | mg total Phenolic Compounds predicted /g Blueberry | Relative error of prediction (%) |
|---|---|---|---|---|---|---|---|---|---|---|---|---|
| **1** | 50 | 40 | 30 | 0.45 | 2 | 10 | 14.05 | 13.36 | 4.88 | 23.06 | 22.35 | 3.06 |
| **2** | 50 | 40 | 70 | 0.45 | 2 | 10 | 14.72 | 14.81 | 0.62 | 23.54 | 22.80 | 3.15 |
| **3** | 50 | 40 | 30 | 0.45 | 7 | 10 | 14.67 | 14.27 | 2.74 | 22.53 | 22.08 | 2.00 |
| **4** | 50 | 40 | 70 | 0.45 | 7 | 10 | 14.88 | 14.99 | 0.75 | 22.99 | 24.13 | 4.94 |
| **5** | 50 | 40 | 30 | 0.45 | 2 | 20 | 13.90 | 13.49 | 2.92 | 25.72 | 25.14 | 2.27 |
| **6** | 50 | 40 | 70 | 0.45 | 2 | 20 | 16.02 | 16.71 | 4.36 | 25.50 | 25.39 | 0.40 |
| **7** | 50 | 40 | 30 | 0.45 | 7 | 20 | 13.16 | 12.77 | 2.93 | 22.86 | 24.16 | 5.67 |

| | | | | | | | | | | | | |
|---|---|---|---|---|---|---|---|---|---|---|---|---|
| **8** | 50 | 40 | 70 | 0.45 | 7 | 20 | 14.28 | 15.26 | 6.92 | 25.86 | 26.01 | 0.57 |
| **9** | 50 | 10 | 50 | 0.2 | 2 | 15 | 13.20 | 12.60 | 4.56 | 23.67 | 23.90 | 0.98 |
| **10** | 50 | 70 | 50 | 0.2 | 2 | 15 | 13.44 | 12.47 | 7.21 | 25.24 | 24.67 | 2.28 |
| **11** | 50 | 10 | 50 | 0.7 | 2 | 15 | 13.78 | 13.03 | 5.49 | 24.70 | 24.58 | 0.50 |
| **12** | 50 | 70 | 50 | 0.7 | 2 | 15 | 13.81 | 13.77 | 0.31 | 23.79 | 23.32 | 1.97 |
| **13** | 50 | 10 | 50 | 0.2 | 7 | 15 | 11.56 | 11.08 | 4.16 | 20.81 | 21.88 | 5.15 |
| **14** | 50 | 70 | 50 | 0.2 | 7 | 15 | 9.61 | 10.88 | 13.18 | 24.56 | 24.08 | 1.96 |
| **15** | 50 | 10 | 50 | 0.7 | 7 | 15 | 13.63 | 14.08 | 3.28 | 24.33 | 25.51 | 4.85 |
| **16** | 50 | 70 | 50 | 0.7 | 7 | 15 | 13.93 | 14.75 | 5.89 | 26.53 | 25.69 | 3.17 |
| **17** | 25 | 40 | 30 | 0.2 | 4.5 | 15 | 16.90 | 17.03 | 0.73 | 22.23 | 21.35 | 3.93 |
| **18** | 75 | 40 | 30 | 0.2 | 4.5 | 15 | 14.50 | 15.68 | 8.09 | 24.22 | 21.80 | 9.96 |
| **19** | 25 | 40 | 70 | 0.2 | 4.5 | 15 | 14.95 | 12.58 | 15.84 | 20.04 | 20.75 | 3.56 |
| **20** | 75 | 40 | 70 | 0.2 | 4.5 | 15 | 19.24 | 19.65 | 2.11 | 25.55 | 23.81 | 6.81 |
| **21** | 25 | 40 | 30 | 0.7 | 4.5 | 15 | 15.63 | 15.62 | 0.02 | 23.97 | 23.86 | 0.45 |
| **22** | 75 | 40 | 30 | 0.7 | 4.5 | 15 | 17.99 | 16.96 | 5.72 | 19.54 | 20.68 | 5.80 |
| **23** | 25 | 40 | 70 | 0.7 | 4.5 | 15 | 15.37 | 15.60 | 1.49 | 23.59 | 24.16 | 2.43 |
| **24** | 75 | 40 | 70 | 0.7 | 4.5 | 15 | 24.88 | 25.35 | 1.87 | 20.86 | 23.58 | 13.08 |
| **25** | 50 | 10 | 30 | 0.45 | 4.5 | 10 | 14.62 | 14.07 | 3.73 | 17.09 | 18.57 | 8.69 |
| **26** | 50 | 70 | 30 | 0.45 | 4.5 | 10 | 15.00 | 14.98 | 0.17 | 21.08 | 22.06 | 4.63 |
| **27** | 50 | 10 | 70 | 0.45 | 4.5 | 10 | 13.42 | 15.37 | 14.51 | 20.54 | 20.43 | 0.53 |
| **28** | 50 | 70 | 70 | 0.45 | 4.5 | 10 | 15.40 | 15.86 | 2.99 | 23.94 | 22.69 | 5.22 |
| **29** | 50 | 10 | 30 | 0.45 | 4.5 | 20 | 13.97 | 13.81 | 1.12 | 22.71 | 23.40 | 3.08 |
| **30** | 50 | 70 | 30 | 0.45 | 4.5 | 20 | 14.52 | 13.87 | 4.49 | 22.54 | 22.09 | 1.98 |
| **31** | 50 | 10 | 70 | 0.45 | 4.5 | 20 | 17.15 | 16.88 | 1.59 | 25.49 | 25.07 | 1.65 |
| **32** | 50 | 70 | 70 | 0.45 | 4.5 | 20 | 16.27 | 16.52 | 1.56 | 23.46 | 22.53 | 3.94 |
| **33** | 25 | 10 | 50 | 0.45 | 2 | 15 | 11.60 | 12.27 | 5.83 | 21.68 | 21.99 | 1.44 |

| | | | | | | | | | | | | | |
|---|---|---|---|---|---|---|---|---|---|---|---|---|---|
| **34** | 75 | 10 | 50 | 0.45 | 2 | 15 | 17.84 | 18.69 | 4.79 | 23.36 | 23.18 | 0.77 |
| **35** | 25 | 70 | 50 | 0.45 | 2 | 15 | 12.98 | 13.45 | 3.56 | 19.59 | 21.15 | 7.96 |
| **36** | 75 | 70 | 50 | 0.45 | 2 | 15 | 17.46 | 18.14 | 3.88 | 22.16 | 23.53 | 6.20 |
| **37** | 25 | 10 | 50 | 0.45 | 7 | 15 | 14.55 | 13.39 | 7.97 | 25.27 | 23.29 | 7.82 |
| **38** | 75 | 10 | 50 | 0.45 | 7 | 15 | 17.05 | 17.10 | 0.30 | 22.96 | 20.79 | 9.45 |
| **39** | 25 | 70 | 50 | 0.45 | 7 | 15 | 15.86 | 14.49 | 8.61 | 23.11 | 23.89 | 3.40 |
| **40** | 75 | 70 | 50 | 0.45 | 7 | 15 | 17.67 | 16.48 | 6.74 | 22.27 | 22.57 | 1.36 |
| **41** | 25 | 40 | 50 | 0.2 | 4.5 | 10 | 14.58 | 15.37 | 5.38 | 19.19 | 19.29 | 0.52 |
| **42** | 75 | 40 | 50 | 0.2 | 4.5 | 10 | 20.73 | 19.68 | 5.08 | 17.24 | 19.01 | 10.27 |
| **43** | 25 | 40 | 50 | 0.7 | 4.5 | 10 | 14.84 | 13.28 | 10.52 | 23.76 | 23.00 | 3.19 |
| **44** | 75 | 40 | 50 | 0.7 | 4.5 | 10 | 18.41 | 20.28 | 10.15 | 20.54 | 19.09 | 7.05 |
| **45** | 25 | 40 | 50 | 0.2 | 4.5 | 20 | 14.59 | 14.13 | 3.15 | 20.74 | 20.34 | 1.90 |
| **46** | 75 | 40 | 50 | 0.2 | 4.5 | 20 | 17.38 | 15.54 | 10.64 | 21.53 | 24.13 | 12.11 |
| **47** | 25 | 40 | 50 | 0.7 | 4.5 | 20 | 16.18 | 17.83 | 10.17 | 22.47 | 22.55 | 0.36 |
| **48** | 75 | 40 | 50 | 0.7 | 4.5 | 20 | 21.30 | 21.92 | 2.87 | 24.65 | 22.70 | 7.89 |
| **49** | 50 | 40 | 50 | 0.45 | 4.5 | 15 | 15.58 | 16.21 | 4.04 | 25.82 | 25.46 | 1.43 |
| **50** | 50 | 40 | 50 | 0.45 | 4.5 | 15 | 16.28 | 16.21 | 0.40 | 25.34 | 25.46 | 0.47 |
| **51** | 50 | 40 | 50 | 0.45 | 4.5 | 15 | 17.09 | 16.21 | 5.11 | 25.20 | 25.46 | 0.99 |
| **52** | 50 | 40 | 50 | 0.45 | 4.5 | 15 | 15.51 | 16.21 | 4.55 | 25.63 | 25.46 | 0.67 |
| **53** | 50 | 40 | 50 | 0.45 | 4.5 | 15 | 15.21 | 16.21 | 6.59 | 24.96 | 25.46 | 1.97 |
| **54** | 50 | 40 | 50 | 0.45 | 4.5 | 15 | 16.60 | 16.21 | 2.33 | 25.78 | 25.46 | 1.25 |
